# Rapid and precise diagnosis of pneumonia coinfected by *Pneumocystis jirovecii* and *Aspergillus fumigatus* assisted by next-generation sequencing in a patient with systemic lupus erythematosus: a case report

**DOI:** 10.1186/s12941-021-00448-5

**Published:** 2021-06-26

**Authors:** Yili Chen, Lu Ai, Yingqun Zhou, Yating Zhao, Jianyu Huang, Wen Tang, Yujian Liang

**Affiliations:** 1 Department of Laboratory Medicine, The First Affiliated Hospital of Sun Yat-Sen University, Guangzhou, 510080 Guangdong China; 2 Department of Laboratory Medicine, The First People’s Hospital of Nanning, Nanning, 530022 Guangxi China; 3 Department of Pediatric Intensive Care Unit, The First Affiliated Hospital, Sun Yat-Sen University, Guangzhou, 510080 China

**Keywords:** *Pneumocystis jirovecii*, *Aspergillus fumigatus*, Next generation sequencing, Case report

## Abstract

**Background:**

*Pneumocystis jirovecii* and *Aspergillus fumigatus*, are opportunistic pathogenic fungus that has a major impact on mortality in patients with systemic lupus erythematosus. With the potential to invade multiple organs, early and accurate diagnosis is essential to the survival of SLE patients, establishing an early diagnosis of the infection, especially coinfection by *Pneumocystis jirovecii* and *Aspergillus fumigatus*, still remains a great challenge.

**Case presentation:**

In this case, we reported that the application of next -generation sequencing in diagnosing *Pneumocystis jirovecii* and *Aspergillus fumigatus* coinfection in a Chinese girl with systemic lupus erythematosus (SLE). Voriconazole was used to treat pulmonary aspergillosis, besides sulfamethoxazole and trimethoprim (SMZ-TMP), and caspofungin acetate to treat *Pneumocystis jirovecii* infection for 6 days. On Day 10 of admission, her chest radiograph displayed obvious absorption of bilateral lung inflammation though the circumstance of repeated fever had not improved. Unfortunately, the patient discharged from the hospital since the financial burden, and during the follow-up, it was documented the patient died within one week after discharge.

**Conclusions:**

This successful application of the next generation sequencing assisting the rapid diagnosis of *Pneumocystis jirovecii* and *Aspergillus fumigatus* coinfection provides a new perspective in the clinical approach against the systematic fungi infections and highlights the potential of this technique in rapid etiological diagnosis.

## Background

Systemic lupus erythematosus (SLE) is a chronic multi-systemic autoimmune disease that involve any organ of the body, and infection remains an important cause of morbidity and mortality in patients with SLE [1]. The incidence of fungal infection, especially *Aspergillus* infection, has increased significantly in patients with SLE, which has become one of the main causes of death in critically ill patients with SLE [2, 3]. *Pneumocystis jiroveciiis*, the causative agent of *Pneumocystis* pneumonia (PCP), which is a common and often life-threatening opportunistic infection in both HIV- infected and non- HIV immunocompromised patients. Compared with the HIV-infected population, the number of the non-HIV immunocompromised patients including SLE patients with PCP is much higher, and the in-hospital mortality reaches from 24 to 67% [4–8].

Early diagnosis and appropriate treatment is essential to the survival of SLE patients, but establishing an early diagnosis of the infection, especially coinfection by *Pneumocystis jirovecii* and *Aspergillus fumigatus*, still remains a great challenge [9]. *Pneumocystis* coinfections with other microorganisms are less frequently described and only sparse reports of combined PCP and *Aspergillus fumigatus* infections exist in the literature, especially in the non- HIV patients [10]. On the other hand, coinfections of *Pneumocystis jirovecii* and other microorganisms are considered as index of poor prognosis in immunocompromised patients, especially when there is invasive aspergillosis [11]. Under the circumstances, a revolutionary technology called next-generation sequencing (NGS) emerged in 2005 has become an attractive alternative method for broad-based pathogen discovery due to the rapid turnaround time and high accuracy. In this manuscript, we described an interesting case of pulmonary coinfection by *Pneumocystis jirovecii* and *Aspergillus fumigatus* which was rapid diagnosed by next-generation sequencing within 24 h in SLE patients, and to our knowledge it is the first documented case in SLE patients.

## Case presentation

A 14-year-old girl was admitted to hospital (The First Affiliated Hospital of Sun Yat-sen University, Guangzhou, China) due to fever and cough for 6 days. With no obvious cause, she developed fever and occasional dry cough without dyspnea and headache. Four months ago, she presented to the pediatric ward for edema and foam urine, and further examination demonstrated that antinuclear antibodies (ANA) 181.65 U/ml (normal range, 0–12 U/ml), anti-double-stranded DNA antibody (dsDNA) 643.19 IU/ml (normal range, 0–30 IU/ml), Complement 3 0.12 g/L (normal range, 0.79–1.17 g/L), Complement 4 0.03 g/L (normal range, 0.17–0.31 g/L), skin rash, urine sugar and protein positive, which made the diagnosed of systemic lupus erythematosus (SLE) established according to American College of Rheumatology (ACR) criteria. She was administered combined methylprednisolone 60 mg/d, hydroxychloroquine, and immunosuppressant tacrolimus.

On admission (Day 1), she got hyperthermia (38.8 ℃). Upon physical examination, her blood pressure was 129/84 mmHg, pulse rate 96/minute and respiratory rate 27/min. It was characterized by hemorrhagic spots and purple streaks scattered all over the skin, and depressed edema of both lower extremities. Initial laboratory evaluation revealed leukocytosis (17.50 × 10^9^/L, 86% neutrophils) with high serum C-reactive protein (36.83 mg/L, normal range 0–10 mg/L). Platelet count was normal (189 × 10^9^/L). Elevate IL-6 level was 21.43 pg/mL (normal range 0–5.3 pg/mL) with procalcitonin level at 0.23 mg/L (normal range 0–0.05 mg/L). Arterial blood gases were: pO2 58.7 mmHg, pCO2 36.8 mmHg, sO2 93.4% and pH 7.45. The additional tests revealed the plasma glucose 12.1 mmol/L (normal range 3.9–6 mmol/L), 2 h plasma glucose after oral glucose tolerance test (OGTT) 15.1 mmol/L (normal range < 7.8 mmol/L), the glycated hemoglobin of 9.90% (normal range 4.4–6.4%) and urine glucose positive, which suggested the diagnosis of diabetes.

**Fig. 1:**
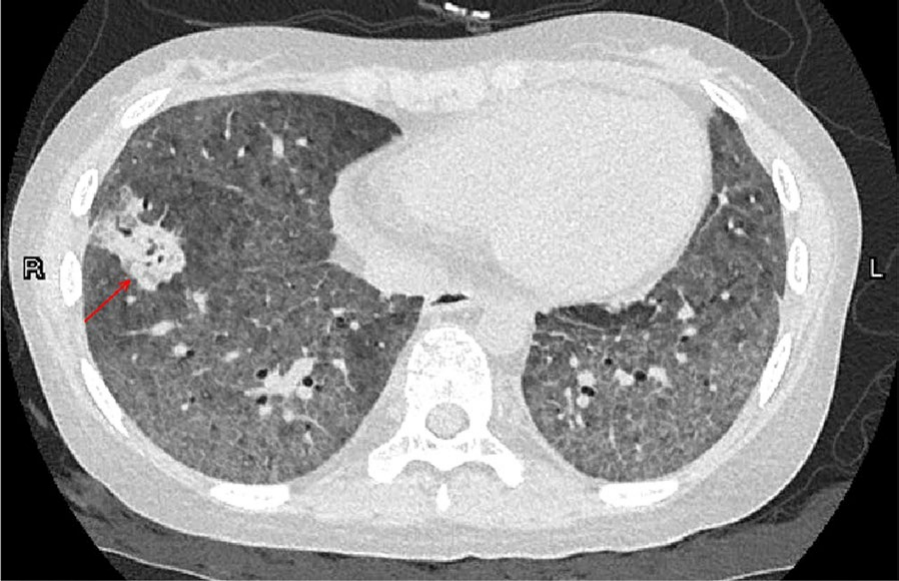
The chest computed tomography (CT) imaging revealed diffuse ground glass changes in the bilateral lungs, partial consolidation(red arrow) and mild bronchiectasis of the anterior basal ganglia in the right lower lobe, and bullae formation in apical segment of upper lobe of left lung

Initial antibiotherapy with intravenous drip of meropenem 1.0 g every 8 h was started. However, the oxygenation of the patient continued to deteriorate, with recurrent fever. On Day 4 the chest computed tomography (CT) imaging revealed: diffuse ground glass changes in the bilateral lungs, partial consolidation and mild bronchiectasis of the anterior basal ganglia in the right lower lobe, and bullae formation in apical segment of upper lobe of left lung (Fig. 1). Without any clinical respiratory improvement, another microbiological tests were performed. Serum β-(1,3)-d glucan was elevated (436.1 pg/mL) with galactomannan (GM) level at 0.4 (cutoff value of 0.90).

**Fig. 2:**
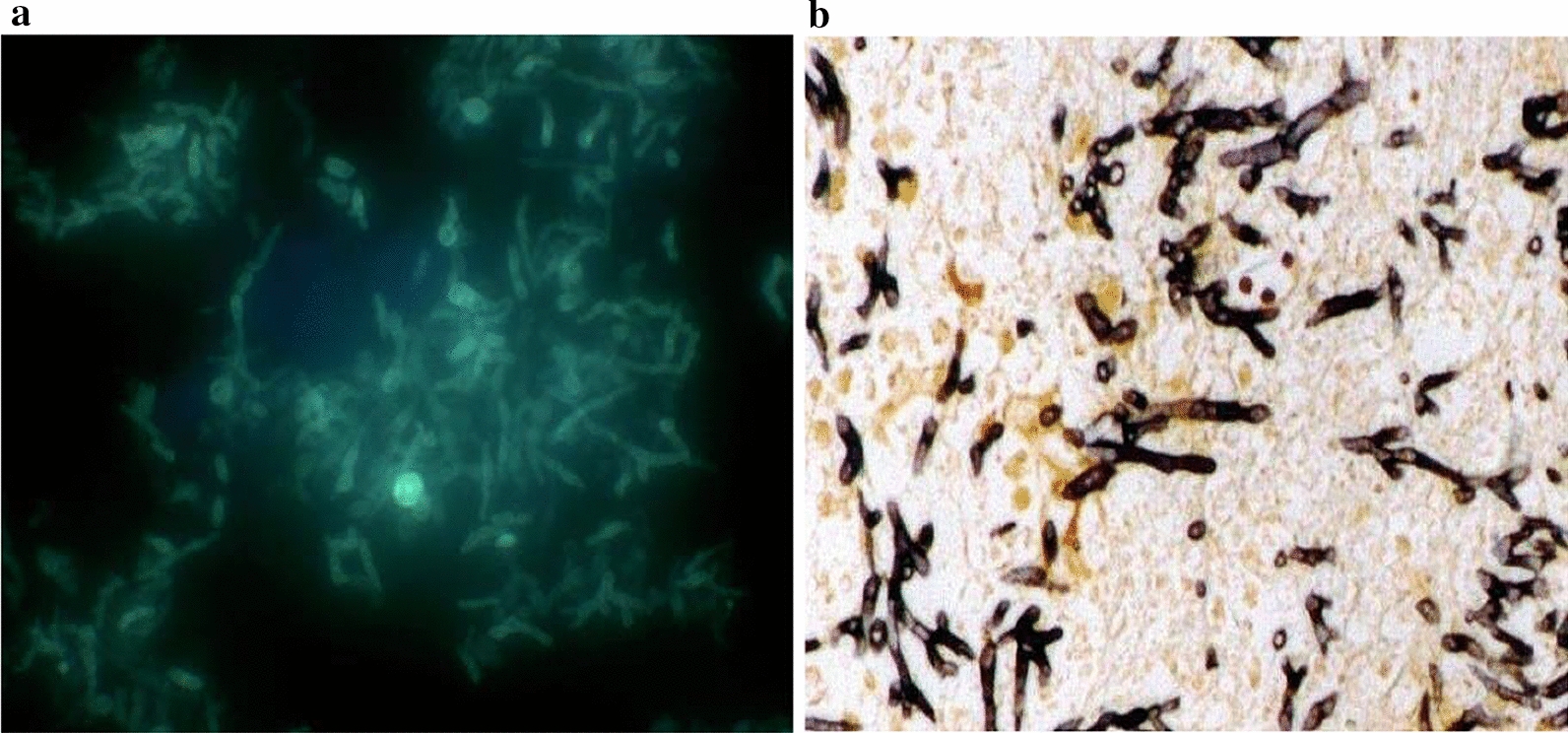
The hyphaes was detected with rapid fluorescence detection of Aspergillus (**a**), and hexamine silver staining on the BALF demonstrated positive (**b**)

**Fig. 3:**
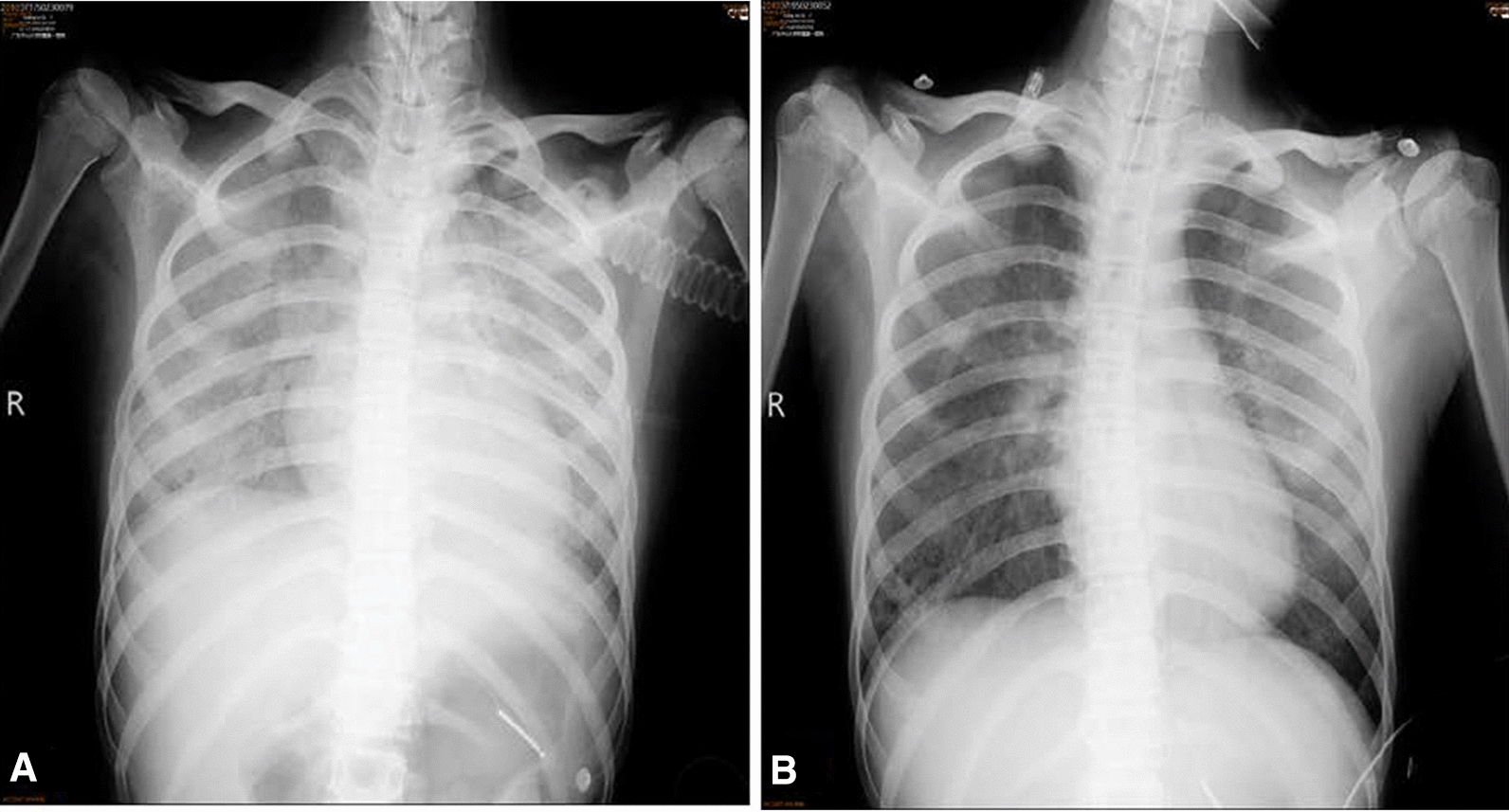
Compared with the X-ray image on admission day (**A**), the chest radiograph image displayed obvious absorption of bilateral lung inflammation after treatment against *Pneumocystis jirovecii* and *Aspergillus fumigatus *(**B**)

On day 5, bonchoalveolar lavage fluid (BALF) were obtained for microbiological examination. Next generation sequencing the BALF specimen was performed, which identified numerous *Aspergillus fumigatus* nucleotide sequences (8, 833 of 22,887,333 cover rate 0.038%) and *Pneumocystis jirovecii* (109,593 of 22,887,333, cover rate 0.48%) within 24 h (Table 1). Initial antibiotherapy with intravenous drip of meropenem 1.0 g every 8 h was started. However, the oxygenation of the patient continued to deteriorate, with recurrent fever. Contemporary inspection results show that BALF galactomannan (GM) was strong positive (4.90, cutoff value of 0.90), and the hyphaes was detected with rapid fluorescence detection as *Aspergillus* (Fig. 2a). Moreover, we performed hexamine silver staining on the BALF and it demonstrated positive confirming the existence of *Pneumocystis* infection (Fig. 2b). According to the EORTC/MSG definition [12], based on host factors, clinical features, and microbiological evidence, this patient was diagnosed as the probable invasive pulmonary aspergillosis (IPA). Therefore on Day 5, treatment against coinfection of *Pneumocystis jirovecii* and *Aspergillus fumigatus* was started. Antibiotic therapy was changed to voriconazole to treat pulmonary aspergillosis, besides sulfamethoxazole and trimethoprim (SMZ-TMP), and caspofungin acetate to treat *Pneumocystis jirovecii* infection. Hormone methylprednisolone was reduced to 20 mg per day, and hydroxychloroquine was discontinued. On Day 8, *Aspergillus fumigatus* was isolated on Sabouraud’s agar. On Day 10, chest radiograph displayed obvious absorption of bilateral lung inflammation (Fig. 3), though the recurrent of fever. Unfortunately, considering the severity of the patient's condition, difficulty in immune reconstitution and poor prognosis, the patient discharged from the hospital, since her family chose abandonment of treatment due to financial burden. During the follow-up, it was documented the patient died within one week after discharge.

**Table 1:** NGS report of the microorganism in BALF

Genus		Species	
Name	Sequence number^a^	Name	Sequence number^a^
Pneumocystis	110174	*Pneumocystis jirovecii*	109593
Aspergillus	8891	*Aspergillus fumigatus*	8833

^a^The sequence number of the strict comparison of the microorganism detected at the level of genus/species

## Discussion and conclusion

Systemic lupus erythematosus (SLE) is a chronic autoimmune disease involving multiple organs and system. Immune system disorder, T cell and B cell dysfunction, the use of corticosteroids and immunosuppressive agents result in low immunity and prone to infection in SLE patients. At the mean time, infection can also aggravate the activity of SLE [13]. The infection site in SLE patients are widely distributed, and the most common infection sites were the lungs, followed by upper respiratory tracts and urinary tracts [14]. A retrospective review performed in 3,831 hospitalized SLE patients revealed that in SLE death cases mainly dying from infection, *Aspergillus fumigatus* and *Pneumocystis carinii* were the two most commonly infected pathogens, and *Cytomegalovirus* was a frequent pathogen of polyinfection [15]. It was worth noting that polyinfections were more frequent than single pathogen infection (60.5 vs. 39.5%), including common bacteria, fungal infection, and cytomegalovirus, simultaneously the mortality was significantly higher.The mix infections caused by *Aspergillus fumigatus* as well as *Pneumocystis jirovecii* are serious and often lethal diseases in severely immunocompromised patients [16]. Moreover the incidence of IPA and PCP increases with the extent of the underlying or therapeutic immunosuppression, and, especially for IPA, has a major impact on morbidity and mortality [17].

An enhancement of clinical awareness, a rapid and accurate diagnostic, and precise management is crucial for early treatment in immunocompromised SLE patients. However in severely immunocompromised patients, simultaneous or secondary infections in *Pneumocystis jirovecii* pneumonia are mostly caused by bacterial pathogens, whereas fungal pathogens, especially *Aspergillus fumigatus*, are rarely diagnosed [18]. In this case, the etiological diagnosis of the SLE patient depended on timely collection of BALF samples, rapid and accurate next generation sequencing technology, as well as combination with microscopic examination, culture and serological diagnosis. Although the final outcome of the patient after discharge was death, a reasonable anti-infection treatment started after the laboratory diagnosis was clear. One week after treatment, imaging examination indicated that the patient's condition was once controlled, suggesting that early, rapid and accurate laboratory diagnosis played a key role in the diagnosis and treatment of immunosuppressive host pulmonary infection.

Nowadays the diagnosis of *Pneumocystis* pneumonia (PCP) is usually achieved by examining stained smears of BALF for the presence of the organism, and the cloning and sequencing of *Pneumocystis jiroveciiis* provide an alternative method. Moreover, the diagnosis of IPA relies mainly on tissue culture and morphological analysis, which may bring drawbacks due to the limited culture positive rate and is sometimes time consuming [19]. The introduction of biomarkers such as galactomannan (GM) and polymerase chain reaction (PCR) assays make big difference in the diagnosis of IPA [20]. With advantages of high sensitivity, low cost and fast detection, however, PCR method requires the physician to raise a few suspicious pathogens prior to examination and is usually restricted to only limited range of pathogens. The radiographic evaluation still has difficulty to make the radiographic diagnosis of combined IPA and PCP. Therefore, these methods are not best applicable in certain clinical situations such as polyinfection in SLE patients.

Next-generation sequencing (NGS) or massively parallel sequencing, a method of simultaneously sequencing millions of fragments of DNA, has been rapidly adopted in the clinical laboratory. Under such circumstances, the implementation of NGS in clinical field enabled a fast and comparably accurate diagnostic tool for physicians,and most importantly, it does not require a predefined range of suspicious pathogens.NGS is an assay that can sequence the entire DNA/RNA of a sample, and this process does not need any primers or probes. It holds the promise of identifying most of the pathogens. Furthermore, NGS can generate billions of DNA/RNA sequences per run, and this enables metagenomic analysis. The principles of NGS in infectious diseases are composed of three main procedures, the identification of the nucleotides in the targeted samples, the comparison of these nucleotides against the catalogue of causative agents, and the decision-making progress whether the acquired sequences points to the possible etiological hypothesis [21]. The first literature applying NGS in the diagnosis of infection was published in 2014, in which leptospira sequence were detected in the spinal fluid of neuroleptospirosis patient [22]. Since then, multiple studies have reported the use of NGS in central nervous system, bloodstream, and respiratory infections [23–26]. The advantage of NGS is its potential to detect multiple pathogens simultaneously in one test while immunosuppressed patients are more likely to be the largest beneficiaries of NGS because they often have coinfections [27]. The major challenge of clinical use of NGS is how to interpret the results and determine whether the microorganism whose sequences are identified is truly the causative pathogen. The nature of NGS tends to detect all nucleotide sequences not only from the samples but also those acquired from the contamination during clinical procedures or laboratory processing, and thus it is challenging to discriminate causative pathogens from normal microbes and environmental contaminants. A microbe found by NGS is usually considered a causative agent based in part or in total on the following clues or evidence: the coverage rate (species level) is significantly greater than that of any other microbe or that in the control materials, or the mapping read number is at the top of the microbes list [28]; the result is confirmed by traditional techniques such as culture, serological testing, PCR, Sanger sequencing, and histopathology; the sequence or read number or pathogen load is significantly reduced during the course of treatment [29]; and the patient improved after targeted antimicrobial agent treatment [28, 30, 31].

Detecting specific microorganisms by NGS is just the first step. However, not all microorganisms determined by NGS are relevant to infectious diseases, such as oral colonizing bacteria (e.g., *Streptococcus parasanguinis*, *Streptococcus mitis*, and *Prevotella melaninogenica*). Taken together, clinicians should identify the real pathogen according to both NGS and other examinations.

In our case, NGS identified *Pneumocystis jirovecii* and *Aspergillus fumigatus* the causative agent within 24 h. The sequencing data, which was in consistent with the patient’s biological and morphologic evidence, clinical and radiological features, assisted clinical physicians in approaching the diagnosis of mixed infection apace and effectively. With the help of NGS on the rapid diagnosis, the patient’s chest radiograph displayed obvious absorption of bilateral lung inflammation after 6 days of target treatment. The limitation of this case study included lacking a validated quantification method to interpret the significance of the NGS result.

In summary, we report a case of a SLE patient with simultaneous *Pneumocystis* pneumonia and invasive aspergillosis, and as we know it is the first documented case in SLE patients. This case highlights the challenges in the diagnosis and management of this rare dual infection and some of its devastating consequences.The introduction of next-generation sequencing applied to pathogen identification dramatically improves the ability to identify poly-infection that occasionally cause disease but lethal. It helps clinicians to make a rapid and precise diagnosis of infectious diseases in the near future.

## Data Availability

Data and materials of this report are publicly available.

## Abbreviations

SLE: Systemic lupus erythematosus
PCP: Pneumocystis pneumonia
IPA: Invasive Pulmonary Aspergillosis
NGS: Next-generation sequencing
BALF: Broncho alveolar lavage fluid
EORTC/MSG: The European Organization for Research and Treatment of Cancer and the Mycoses Study Group
